# Editorial Notices

**Published:** 1875-07

**Authors:** 


The Nashville Medical and Surgical Journal for June brings us intelligence of the retirement of the senior editor, Dr. William K. Bolling, who founded that Journal a quarter of a century ago, and has so long and so ably sustained it by his pen, which, though caustic and trenchant at times, is always ready and vigorous. We wish for him in his retirement all the peaceful and quiet enjoyment of life which ought to reward the long years of past journalistic labor and vexation.

---

The New Orleans Medical and Surgical Journal has passed into the hands of new publishers—Messrs. Seymour & Stevens, 96 and 98 Commerce Street, New Orleans. It is very ably edited by Dr. Samuel M. Bemiss, and deserves a much more liberal patronage than it has as yet received at the hands of the profession of the country. It is issued bi-monthly at $5 per year.

